# New insights into the pathogenesis of IgA nephropathy

**DOI:** 10.1007/s00467-017-3699-z

**Published:** 2017-06-17

**Authors:** See Cheng Yeo, Chee Kay Cheung, Jonathan Barratt

**Affiliations:** 1 Department of Renal Medicine, Tan Tock Seng Hospital, Singapore, Singapore; 2 Department of Infection, Immunity and Inflammation, University of Leicester, University Road, Leicester, LE1 7RH UK; 3 The John Walls Renal Unit, Leicester General Hospital, Leicester, UK

**Keywords:** IgA nephropathy, Pathogenesis, IgA1, *O*-galactosylation, Immune complexes

## Abstract

IgA nephropathy is the most common form of glomerulonephritis in many parts of the world and remains an important cause of end-stage renal disease. Current evidence suggests that IgA nephropathy is not due to a single pathogenic insult, but rather the result of multiple sequential pathogenic “hits”. An abnormally increased level of circulating poorly *O*-galactosylated IgA1 and the production of *O*-glycan-specific antibodies leads to the formation of IgA1-containing immune complexes, and their subsequent mesangial deposition results in inflammation and glomerular injury. While this general framework has formed the foundation of our current understanding of the pathogenesis of IgA nephropathy, much work is ongoing to try to precisely define the genetic, epigenetic, immunological, and molecular basis of IgA nephropathy. In particular, the precise origin of poorly *O-*galactosylated IgA1 and the inciting factors for the production of *O*-glycan-specific antibodies continue to be intensely evaluated. The mechanisms responsible for mesangial IgA1 deposition and subsequent renal injury also remain incompletely understood. In this review, we summarize the current understanding of the key steps involved in the pathogenesis of IgA nephropathy. It is hoped that further advances in our understanding of this common glomerulonephritis will lead to novel diagnostic and prognostic biomarkers, and targeted therapies to ameliorate disease progression.

## Introduction

Since its first description, *les dépôts intercapillaires d’IgA-IgG* (intercapillary deposits of IgA-IgG), by Berger and Hinglais in 1968 [1], IgA nephropathy (IgAN) continues to be recognized as the most common form of glomerulonephritis in many parts of the world [2–6]. While the disease runs a relatively benign course in the majority of patients, up to 40% of patients progress to end-stage renal disease (ESRD) over the course of 30 to 40 years. Over the past two decades, significant advances have been made in our understanding of the pathogenesis of IgAN. It is now widely accepted that IgAN does not arise from a single pathogenic “hit”, but rather arises as a consequence of multiple sequential but distinct pathogenic “hits”: principally, an increased level of poorly *O*-galactosylated IgA1 glycoforms, production of *O*-glycan-specific antibodies, and the formation of IgA1-containing immune complexes. The resultant deposition of IgA1-containing immune complexes in the glomerular mesangium drives cellular proliferation and overproduction of extracellular matrix, cytokines and chemokines, culminating in glomerular injury. This current concept of the pathogenesis of IgAN has been referred to as the “multi-hit” hypothesis [7].

Much of the work on the pathogenesis of IgAN has centered on understanding the nature of circulating IgA1-containing immune complexes in IgAN and this has been driven by two key clinical observations. Firstly, IgAN may recur in transplanted kidneys in patients with IgAN, and secondly, that clearance of IgA deposits may occur in transplanted IgAN kidneys that have been inadvertently transplanted into recipients without IgAN [8–10]. These two observations indicate that the initiating pathogenic insult in IgAN must arise outside of the kidney. As we will review below, there are striking changes in the physicochemical properties of circulating IgA1 molecules and development of circulating *O*-glycan-specific antibodies in IgAN, which correlate with the composition of mesangial IgA deposits isolated from glomeruli in IgAN.

Another well-recognized clinical observation is that although mesangial IgA deposition is diffuse and global in IgAN, there is significant heterogeneity in both the pathological response to this deposition, which may be focal and segmental, and the corresponding clinical course. The sole criterion for the diagnosis of IgAN is the presence of dominant or co-dominant IgA deposits in the glomerular mesangium on kidney biopsy. Yet, the marked heterogeneity in clinical presentation, clinical course and pathological changes in IgAN is striking. It has been suggested that this heterogeneity likely reflects the varied influence of genetic and environmental factors on a host of complex pathogenic mechanisms that modulate the disease phenotype in different individuals and populations. An alternative explanation that has been proposed is that IgAN may not be a “single disease” but rather a group of distinct diseases sharing a final common pathway of mesangial IgA deposition [11]. This is an important consideration when reviewing conflicting data within the IgAN field, as it may be that authors are describing different disease processes and responses to treatment in subtly different diseases.

Another observation worthy of consideration ahead of any review of IgAN is the fact that sub-clinical mesangial IgA deposition is a relatively common finding in the general population, and in particular in Asian populations. In autopsy series and allograft biopsy series, IgA deposition without overt clinical disease has been observed in up to 16% of subjects [12–14]. It remains unclear whether these sub-clinical IgA deposits are biochemically different and immunologically inert, or if inherent factors in the affected kidneys prevent the propagation of pathogenic pathways and glomerular injury. Importantly, these observations suggest that the mechanisms responsible for inducing glomerular injury in IgAN are distinct from those responsible for mesangial deposition of IgA. The natural history following the finding of subclinical mesangial IgA deposition, and whether this is a risk factor for overt disease in the long term, remains unclear.

At present, the diagnosis of IgAN cannot be made without a kidney biopsy. The varied clinical course of the disease means that many patients will not develop CKD or progress to ESRD. In order to appropriately counsel patients with IgAN on future risks of CKD and ESRD and direct often toxic immunosuppressive drug regimens to those patients most likely to benefit, early identification of patients at greatest risk of progression is essential. Current clinical markers of severity of kidney disease, namely proteinuria, hypertension, and impaired renal function, are non-specific and manifest only when significant (and often irreversible) renal injury and scarring have occurred. A better understanding of the pathogenesis of IgAN is likely to lead to the identification of novel biomarkers to better risk stratify patients and guide treatment choices. Furthermore, current treatment of IgAN remains generic and applicable to many kidney diseases, focusing on modulating downstream immune and inflammatory events, and is not specific to IgAN. It is hoped that advances in our understanding of the pathogenesis of IgAN will identify new pathways amenable to therapeutic manipulation and in this review we give a number of examples of novel therapies currently in phase II trials that have been triggered by a clearer understanding of the molecular basis of IgAN [15, 16]. Here, we will review key pathogenic pathways involved in the development of IgAN.

## IgA1 *O*-galactosylation in IgA nephropathy

A key observation in our understanding of the pathogenesis of IgAN is the increased presence of poorly *O*-galactosylated IgA1 glycoforms in both serum and glomerular immune deposits [17, 18], a finding that has been consistently reproduced in populations of different ethnic and geographic origin [19–22]. These *O*-glycoforms of IgA1 are often referred to in the literature as galactose-deficient IgA1 (gd-IgA1), however, we believe that this is a misnomer, as most of these *O*-glycoforms still carry galactose residues and “deficiency” implies these *O*-glycoforms have been defectively *O*-glycosylated during post-translational modification. On the contrary, we believe poorly *O*-galactosylated IgA1 is the normal *O*-glycosylated form of IgA1 produced at mucosal surfaces and its increased presence in the serum reflects a subtle defect in the mucosal immune system.

**Fig. 1:**
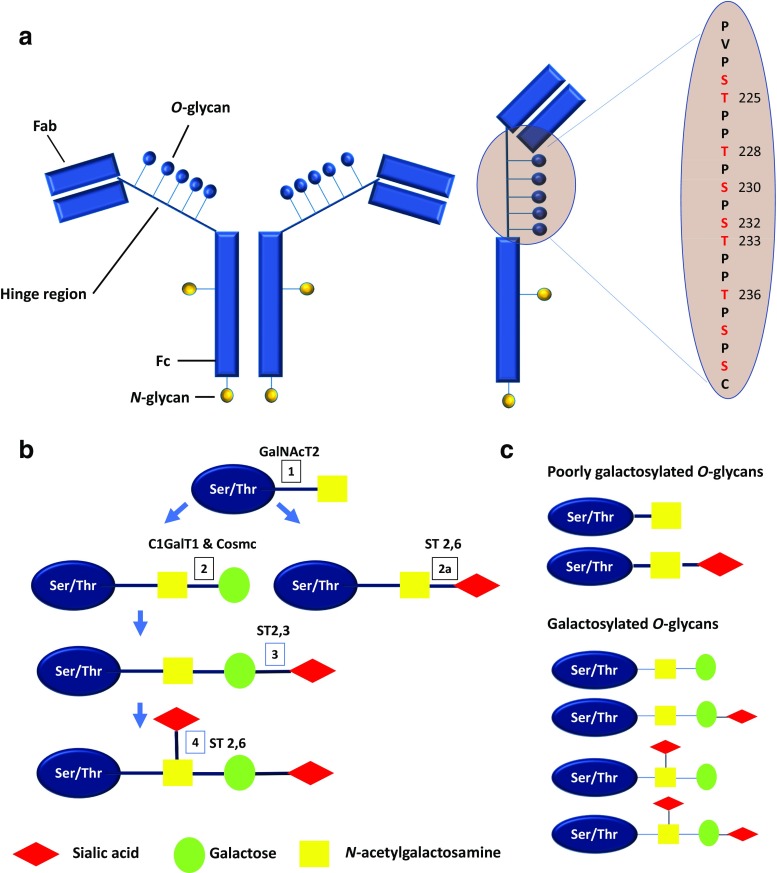
Structure of human IgA1 and its *O*-glycans. **a** IgA1 has an extended hinge region that contains between 3 and 6 *O*-glycans attached to serine or threonine residue between position 225 to 236 (IgA1 with five *O*-glycans per hinge region is shown). **b** Glycosylation of IgA1 is mediated by stepwise co−/post-translational modifications. First,* N*-acetylgalactosamine (GalNAc) is added to serine/threonine residue by activity of* N*-acetylgalactosaminyl-transferase (GalNAcT2) (step 1). Next, a galactose moiety is added to GalNAc by core 1 beta 1, 3-galactosyltransferase (C1GalT1) and core 1 β3GalT-specific molecular chaperone (Cosmc) (step 2). Sialic acid may then be added to the galactose moiety by α2,3 sialytransferase (ST2,3) (step 3) or to the GalNAc moiety by α2,6 sialytransferase (ST2,6) (step 4). Alternatively, sialic acid may be added to GalNAc by ST2,6 before the addition of galactose (step 2a). Notably, sialylated GalNAc (step 2a) cannot be subsequently galactosylated, whereas galactosylated GalNAc may be sialylated at either the GalNAc or galactose moiety, or both (step 3 and/or 4). **c** These steps produce a combination of different *O*-glycoforms of varying degree of galactosylation and sialylation. The relative proportion of poorly galactosylated IgA1 is increased in IgAN

Human IgA consists of two subclasses: IgA1 and IgA2, but only IgA1 is present in the mesangial deposits of IgAN [23]. IgA1 has an 18-amino acid extended hinge region between the first and second constant domains of the α heavy chain where *O*-glycans chains may attach to serine or threonine residues (Fig. 1). Although there are up to nine possible serine/threonine sites available for *O*-galactosylation in each α heavy chain, only between 3 and 6 sites may be occupied at any time. *O*-galactosylation of the hinge region occurs through a series of stepwise co/post-translational modifications mediated by a group of enzymes. This process is initiated by the addition of *N*-acetylgalactosamine (GalNAc) via an oxygen atom to a serine or threonine residue on the IgA1-hinge region by the activity of *N*-acetylgalactosaminyl-transferase (GalNAcT2). The *O*-glycan chain may then be extended by galactosylation, where galactose is β1,3 linked to GalNAc to form a disaccharide by the activity of core 1 beta 1, 3-galactosyltransferase (C1GalT1). Interaction between C1GalT1 and its molecular chaperone, core 1 β3GalT-specific molecular chaperone (Cosmc) is necessary for the stability of C1GalT1 during biosynthesis and to prevent protein misfolding. Sialic acid may be attached to the galactose moiety by α2,3 sialytransferase (ST2,3) or be attached directly to GalNAc in an α2,6 linkage, driven by the activity of α2,6 sialytransferase (ST2,6). It has been proposed that sialylation of GalNAc prevents the further addition of galactose and is therefore an important step in IgA1 *O*-galactosylation [24, 25].

All individuals are capable of synthesizing IgA1 with a range of *O*-galactosylated hinge regions. It has been suggested that we have evolved the ability to alter IgA1 hinge region *O*-glycosylation, in particular to reduce *O*-galactosylation of the IgA1 hinge, as a way of counteracting the activity of IgA1 proteases released by bacterial pathogens attempting to circumvent the mucosal IgA immune system. Importantly, in health, serum contains poorly *O*-galactosylated IgA1 glycoforms, indistinguishable from those seen in IgAN, however, in IgAN they comprise a greater proportion of the IgA1 *O*-glycoform circulating pool.

Useful tools for assessing the relative degree of protein glycosylation are lectin-based binding assays (lectins are proteins that bind to specific carbohydrate groups). The most commonly used lectin assay to measure IgA1 *O*-galactosylation uses the lectin *Helix aspersa* agglutinin (HAA), which preferentially binds poorly *O*-galactosylated IgA1 glycoforms (i.e., exposed GalNAc residues). This assay gives a measure of the overall degree of IgA1 *O*-galactosylation in the serum and is determined by the relative amounts of the different *O*-galactosylated IgA1 glycoforms present. Currently, the only way to measure the relative amounts of individual *O*-glycoforms of IgA1 in serum is to undertake mass spectrometry-based analyses, which while highly informative are not suited to large-scale analysis in IgAN cohorts [26].

A significant drawback to the HAA lectin assay is the variability in stability and bioactivity of HAA between batches (the lectin needs to be isolated from the snail *Helix aspersa,* and a recombinant form is not available). This has led to difficulties in comparing results across laboratories. Recently, a novel monoclonal antibody with specificity for the poorly *O*-galactosylated hinge region has been developed and this may provide the basis for a robust ELISA, although further validation of the assay will be required in different cohorts [27].

To explain the molecular basis for the existence of different *O*-glycoforms of IgA1, it has been proposed that the expression and/or activity of the required glycosyltransferases is differentially regulated in subpopulations of IgA1-committed plasma cells. In IgAN, it has been suggested that there might be widespread downregulation of C1GalT1 and/or Cosmc, while others suggest that excessive sialylation of GalNAc by ST2,6 is key in preventing IgA1 *O*-galactosylation [24, 28–30]. It is, however, highly unlikely that a generic defect in *O*-glycosylation of all IgA1 molecules underlies the shift in the complement of serum IgA1 *O*-glycoforms in IgAN. Importantly, *O*-galactosylation of serum IgD (which is also heavily *O*-galactosylated in humans) is not altered in patients with IgAN, suggesting that the decreased *O*-galactosylation of IgA1 in IgAN is not a consequence of an inherent generalized defect of expression or function of galactosylation enzymes in all B cells [31].

A pivotal role for circulating poorly *O*-galactosylated IgA1 in the pathogenesis of IgAN is supported by two studies that showed that the IgA eluted from mesangial deposits was enriched for poorly *O*-galactosylated IgA1 glycoforms [18, 20]. Furthermore, higher serum levels of poorly *O*-galactosylated IgA1 have been shown to be associated with disease progression in IgAN [32].

## Genetic control of IgA1 *O*-galactosylation

There is convincing evidence that genetic factors play a major role in influencing the composition of circulating IgA1 *O*-glycoforms in serum. Up to half of asymptomatic first-degree relatives of patients with both familial and sporadic IgAN have been found to have high levels of poorly *O*-galactosylated IgA1. Studies of familial IgAN cohorts have previously estimated the heritability of poorly *O*-galactosylated IgA1 to be between 54 and 76%, and in a recent study of healthy monozygotic and dizygotic twin pairs using the classic twin model, the hereditability of poorly *O*-galactosylated IgA1 was found to be as high as 80% [33–37]. In contrast, the same studies demonstrated that serum total IgA levels had low heritability, demonstrating that *O*-galactosylation of IgA1 is independent of serum IgA level. In a recently published study, serum levels of poorly *O*-galactosylated IgA1 were found to be associated with a noncoding region of *C1GALT1*, the gene responsible for encoding the C1GalT1 galactosyltransferase. An association with a noncoding region of the gene is consistent with changes in regulation of *C1GALT1*, perhaps in specific microenvironments such as the mucosa, rather than a generic change in the structure of C1GalT1 galactosyltransferase affecting all cells. This association was not restricted to IgAN but was also found in healthy subjects and cases of membranous nephropathy, in both white and Chinese populations, supporting the view that circulating levels of poorly *O*-galactosylated IgA1 are heritable and influenced by genetic variation within the *C1GALT1* gene [38]. These findings have been replicated in a separate cohort [39]. Interestingly, given that IgAN is more prevalent in Chinese compared to white populations, levels of circulating poorly *O*-galactosylated IgA1 in Chinese IgAN cases were lower than in white IgAN cases and indeed were comparable to that seen in a healthy white population, and this corresponded with the low frequency of the identified *C1GALT1 risk* haplotype in the Chinese population. This observation raises questions on the pathogenic importance of changes in IgA1 *O*-galactosylation in different ethnic populations, and whether other pathogenetic mechanisms also act at variable levels.

Epigenetic control of IgA1 *O*-galactosylation is also thought to be important in IgAN. MicroRNAs (miRNA) are endogenous short, noncoding single-stranded RNA molecules that regulate gene expression. Upregulation and overexpression of a specific miRNA (miR-148b) in peripheral blood mononuclear cells (PBMCs) has been associated with a decreased expression of C1GalT1 and production of poorly *O*-galactosylated IgA1. Intriguingly, a binding site for miR-148b has been identified within the recently identified *C1GALT1* risk haplotype (1365G > A polymorphism or rs1047763) supporting further a role for miR-148b in IgAN [40].

## The origin of poorly *O*-galactosylated IgA1 in IgAN

Recognizing that poorly *O*-galactosylated IgA1 plays a pivotal role in the pathogenesis of IgAN, the origin of the responsible B/plasma cells has been the subject of intensive study. The current belief is that the originating B cells undergo activation and programming in the mucosal immune system, however, a significant proportion of the resultant plasma cells eventually reside in the bone marrow rather than in the mucosa, possibly due to defective trafficking during B cell maturation.

There are numerous lines of evidence supporting the mucosal immune system as the source of poorly *O*-galactosylated IgA1-secreting B/plasma cells. Clinically, patients with IgAN not infrequently develop visible hematuria after an upper respiratory tract infection (termed synpharyngitic hematuria) and this is associated with an increase in circulating IgA immune complex levels [41]. Mucosal IgA, unlike systemic IgA, is typically polymeric, of low affinity, and relatively poorly *O*-galactosylated, the physicochemical characteristics typically observed in serum and mesangial IgA in IgAN [42, 43]. IgAN has also been associated with diseases in which mucosal immune responses are abnormal, such as coeliac disease and inflammatory bowel disease. Significantly, recent genome-wide association studies in IgAN have identified susceptibility loci in genes involved in intestinal mucosal immunity [44].

While the importance of the mucosal-kidney axis in IgAN is being increasingly recognized, it remains unclear how alterations in the mucosal immune system lead to an increase in the complement of poorly *O*-galactosylated IgA1 glycoforms in the serum in IgAN. The majority of circulating IgA1 is monomeric, heavily *O*-galactosylated and derived from bone marrow-residing plasma cells. In contrast, mucosally residing plasma cells synthesize IgA1 that is predominantly polymeric, poorly *O*-galactosylated, and secreted onto mucosal surfaces, with little, if any normally entering the circulation. It has been postulated that the increase in poorly *O*-galactosylated serum IgA1 glycoforms in IgAN is the result of misdirected secretion of “mucosal IgA” into the circulation, rather than onto mucosal surfaces. Intriguingly, the numbers of plasma cells that secrete “mucosal IgA” are reduced in the mucosa but numbers are increased at systemic sites, in particular the bone marrow in IgAN [45, 46]. It has therefore been suggested that this misdirected secretion of “mucosal IgA” into the circulation is the result of mucosal-derived B/plasma cells that have mistrafficked to the bone marrow instead of homing back to mucosal surfaces, resulting in release of “mucosal IgA” directly into the systemic circulation. While there is some evidence for defective homing receptor expression by lymphocytes in IgAN, much more work is required to define precisely the pattern of B cell trafficking in IgAN [47–50].

## Dysregulated mucosal IgA production in IgAN

In parallel with the potential mistrafficking of mucosal B/plasma cells, there is also evidence of a subtly dysregulated mucosal immune response to antigen in IgAN. A number of studies have examined immune responses to mucosal antigen challenge in IgAN and the majority have reported exaggerated systemic IgA responses to mucosal antigen challenge [51–56]. There has been increasing interest examining links between alterations in gut permeability, the gut microbiome, and interaction with the mucosal immune system in IgAN, and these studies have recently been reviewed [57, 58]. One mucosal antigen that has attracted particular attention is gliadin, a component of gluten. Mice subjected to a gluten-free diet from birth, and then exposed to a gluten-rich diet, developed increased IgA deposition, with anti-gliadin IgA found in the serum and glomerular deposit eluates [59]. Furthermore, in a recently developed transgenic mouse model that expresses both human IgA1 and human CD89, and develops IgAN spontaneously, in those fed a gluten-free diet for three generations, there was a reduction in mesangial IgA deposition and glomerular inflammatory cell infiltration [60]. Exposure of these mice to gluten led to increased mesangial IgA deposition and formation of anti-gliadin IgA. In a clinical study of IgAN patients given a gluten-free diet, reductions in hematuria, proteinuria, IgA immune complex formation, and anti-gliadin IgA were observed, but there was no difference in the rate of decline in renal function over 4 years of follow-up [61]. Further studies regarding potential links between dietary antigens and IgA immune complex formation are needed.

The molecular basis of regulation of the mucosal immune response, and in particular mucosal B cell programming, in health, and IgAN are complex. Two events thought to be critical are antigen-driven activation of the innate immune response, in particular through ligation of Toll-like receptors (TLR), and B-cell activating factor (BAFF) and a proliferation inducing ligand (APRIL) signaling [62].

TLRs represent an important part of the early innate immune response to invading microbial pathogens and endogenous danger signals via recognition of a diverse range of pathogen-associated molecular patterns (PAMPs) and danger-associated molecular patterns (DAMPs), such as bacterial lipopolysaccharide (LPS), RNAs, and DNAs [63, 64]. TLRs can be found on a diverse range of cells including macrophages and dendritic cells, and the stimulation of TLRs initiates signaling cascades that result in a variety of cellular responses including the production of interferons (IFNs), and pro-inflammatory and effector cytokines that direct the adaptive immune response. B cells also express a variety of TLRs and specifically TLR-4, −9 and −10 have been implicated in IgAN [62, 65, 66]. Expression of mRNA for TLR-4 in circulating PBMCs is increased in children with IgAN and HSP compared to healthy subjects [67]. Exposure to environmental antigens results in an elevated level of TLR-9 and more severe IgA-mediated injury in a murine IgAN model [62], and stimulation of mucosal lamina propria B cells by a TLR-9 ligand containing the CpG-oligodeoxynucleotide (CpG-ODN) bacterial DNA motif induces IgA production [68]. Besides driving mucosal IgA production, TLR activation can also modify glycosyltransferase activity through methylation of the Cosmc gene resulting in reduced activity of C1GalT1, favoring production of poorly *O*-galactosylated IgA1 [66, 69].

BAFF is necessary for B-cell maturation and survival, and levels of BAFF are elevated in many autoimmune diseases and correlate with autoantibody concentration [70–74]. Mice that overexpress BAFF have raised levels of polymeric IgA, and evidence of mesangial IgA deposition [75]. Importantly, this IgA deposition is dependent on activation of the mucosal immune system. In human IgAN, serum BAFF levels are elevated, and are associated with worse renal histopathologic injury (increased mesangial hypercellularity, segmental glomerulosclerosis, and tubular atrophy/interstitial fibrosis) and higher serum creatinine [76]. Tonsillar mononuclear cells (TMCs), which are part of the mucosal-associated lymphoid tissue of Waldeyer’s ring, from IgAN patients exposed to CpG-ODN produce high levels of BAFF and IgA, and this production of IgA can be inhibited by blockade of BAFF signaling [77]. APRIL, another member of the tumor necrosis factor ligand superfamily (TNFSF), which shares common receptors with BAFF, also plays an important role in B cell maturation and survival, and is involved in generation of IgA-secreting plasma cells. Genome-wide association studies in IgAN have identified *TNFSF13* (which encodes APRIL) as a susceptibility locus [78], and this risk variant is associated with high serum levels of IgA in patients with IgAN. Zhai et al. showed that increased levels of APRIL were associated with increased levels of poorly *O*-galactosylated IgA1 and a more severe clinical presentation [69]. In a recent study, Muto et al. demonstrated that tonsillar TLR9 and APRIL levels were elevated and correlated with one another in IgAN, and that TLR-9 stimulation induced APRIL expression in tonsillar B cells [79].

## Therapeutic manipulation of the mucosal immune system and BAFF/APRIL signaling in IgAN

**Fig. 2:**
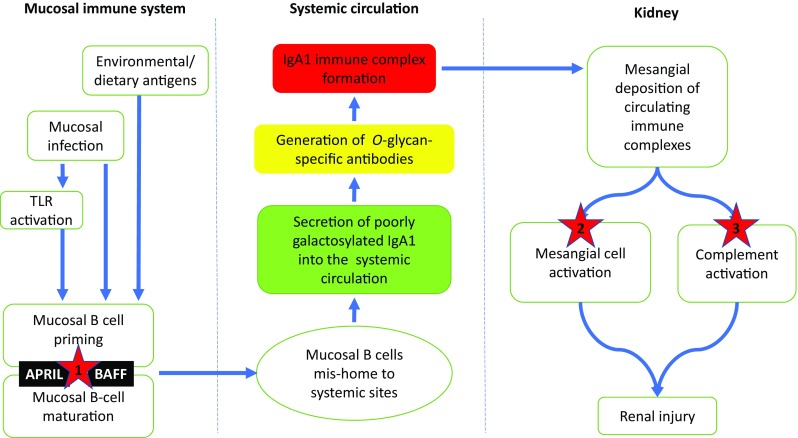
A proposed pathogenic model for IgAN with a focus on potential therapeutic targets. In this model, a dysregulated mucosal immune system results in excessive mucosal IgA-committed B cell proliferation in response to mucosal antigen exposure, mediated in part through excessive BAFF and APRIL signaling. As a result of mis-homing of a proportion of these mucosal B cells to systemic sites mucosal IgA is secreted directly into the circulation resulting in elevated serum levels of polymeric, poorly galactosylated IgA1. In susceptible individuals, *O*-glycan-specific antibodies are formed with the consequent generation of circulating IgA immune complexes, which have a propensity for mesangial deposition. Glomerular accumulation of these IgA immune complexes results in mesangial cell activation, and release of pro-inflammatory and pro-fibrotic mediators, and complement activation. Within this model, there are a number of potential targets (denoted by *) for novel therapeutic agents, many of which are currently under evaluation in clinical trials in IgAN

Taken together, available data suggests that mucosal programming of B cells in IgAN, involving TLRs and BAFF/APRIL, plays a critical role in the development of IgAN. There has, not surprisingly, been great interest in targeting these pathways in IgAN using novel immunomodulatory strategies (Fig. 2) [16].

The NEFIGAN study evaluated targeted immunosuppression of the mucosal immune system using enteric budesonide [80]. This formulation of budesonide is a modified release formulation, designed specifically to deliver budesonide to the ileocecal Peyer’s patches, with minimal systemic exposure and side effects due to first-pass metabolism. In this study, a significant reduction in proteinuria was observed following 9 months of treatment supporting the hypothesis that the mucosal immune system plays an important role in IgAN.

In contrast to targeted mucosal immunomodulation, systemic B cell depletion with rituximab is not effective in IgAN, reinforcing the importance of the mucosal immune system in the pathogenesis of IgAN. In a small open-label randomized controlled trial use of rituximab compared to conventional therapy (without immunosuppression), resulted in more adverse events, did not significantly improve renal function or proteinuria over 1 year and did not reduce serum levels of poorly O-galactosylated IgA1 or anti-IgA1 IgG autoantibodies, despite effective circulating B cell depletion [81].

Blisibimod and atacicept are currently being evaluated in separate phase II studies in IgAN. Both agents target the BAFF and APRIL signaling pathways. Blisibimod is a selective peptibody antagonist of BAFF. Atacicept is a fusion protein containing the extra-cellular, ligand-binding portion of TACI (one of the receptors for BAFF and APRIL) and the modified Fc portion of human IgG, and acts by blocking BAFF and APRIL. Preliminary results from the BRIGHT-SC study, a phase 2, randomized, double-blind, placebo-controlled trial, suggest that subcutaneous blisibimod may prevent worsening of proteinuria in IgAN (ClinicalTrials.gov Identifier: NCT02062684).

Hydroxychloroquine is a potent inhibitor of TLR-9, and to a lesser extent TLR-7 and TLR-8, and inhibits antigen processing and presentation via alkalinization of proteasomes [82, 83]. Given the proposed role of TLRs in the pathogenesis of IgAN, a small paired case-control study has been conducted which described benefit in terms of reduction in proteinuria in treated IgAN patients at 24 weeks follow-up [84]. Further validation in larger randomized studies with longer-term follow-up will be required.

## *O*-glycan-specific autoantibodies and circulating immune complex formation in IgAN

**Fig. 3:**
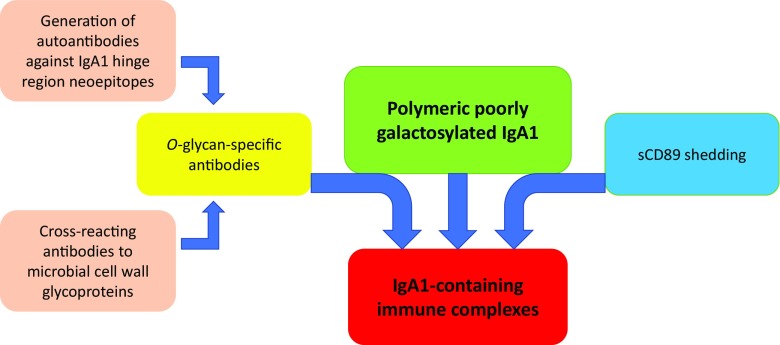
Formation of circulating IgA-immune complexes in IgA nephropathy. Polymeric poorly galactosylated IgA1 molecules form the substrate for immune complex formation. *O*-glycan-specific antibodies: either IgG and IgA1 autoantibodies, or cross-reacting anti-microbial antibodies, bind to the exposed neo-epitopes within the poorly galactosylated IgA1 hinge region. An alternative hypothesis for the formation of circulating IgA immune complexes is that soluble CD89 (sCD89) is shed from myeloid cells in response to polymeric IgA1, and form large circulating IgA1-sCD89 immune complexes

As already eluded to, while the presence of poorly *O*-galactosylated IgA1 is a key observation in IgAN, this finding alone is not sufficient for the development of clinical disease. In in vitro experiments utilizing IgA1 isolated from patients with IgA myeloma, human mesangial cells can be activated by the presence of poorly *O*-galactosylated polymeric IgA1 and IgG/A immune complexes but not by monomeric IgA1 alone [85]. It has therefore been proposed that the formation of circulating immune complexes, perhaps triggered by *O*-glycan-specific antibodies, is necessary for the development of the glomerular injury, and that poorly *O*-galactosylated IgA1 molecules are the substrate for the formation of these immune complexes (Fig. 3). The frequent observation of co-deposits of IgG and occasionally IgM with IgA in the mesangium in IgAN supports the generation of mixed immune complexes in IgAN [86–88]. Serum levels of *O*-glycan-specific antibodies are associated with disease activity and progressive kidney disease, further supporting the role of *O*-glycan-specific antibodies in the pathogenesis of IgAN [89, 90]. It has recently been reported that *O*-glycan-specific IgG antibodies in IgAN contain a specific amino acid sequence, Y1CS3, in the heavy chain variable region, compared with a Y1CA3 sequence in isotype-matched IgG from healthy controls, that the S3 residue is critical for binding to poorly *O*-galactosylated IgA1, and that this substitution is not observed in germline DNA and appears to be a result of a somatic mutation, perhaps influenced by exposure to specific environmental antigens [86, 91].

It has been hypothesized that changes in *O*-galactosylation of the IgA1-hinge region could result in conformational change of the molecule and exposure of novel epitopes within the hinge region. These novel epitopes may then trigger specific *O*-glycan-specific autoantibody production, and/or be recognized by serum antimicrobial antibodies that mistake the IgA1 hinge region *O*-glycans for bacterial or viral cell wall glycoprotein structures (molecular mimicry). One intriguing possibility is that during mucosal infections it is the increased production of antimicrobial mucosal antibodies that heightens the serum *O*-glycan-specific immunoreactivity and drives immune complex formation in IgAN, resulting in a temporary flooding of the glomeruli with IgA immune complexes and short-lived severe glomerular inflammation with development of synpharyngitic hematuria. It is also possible that antimicrobial mucosal antibodies generated at the time of a mucosal infection include poorly galactosylated IgA1, contributing further to both the pool of the target protein and *O*-glycan-specific antibodies in IgAN (Fig. 3).

In support of a pathogenic role for *O*-glycan-specific autoantibody production in IgAN, the strongest signal in genome-wide association studies in IgAN localizes to susceptibility loci on chromosome 6p within the human leucocyte antigen region. These loci are important in determining antigen-processing and presentation, and this association suggests that a dysregulated adaptive immune response may play a role in preferentially presenting poorly *O*-galactosylated IgA1 as a self-antigen and in the permissive production of *O*-glycan-specific antibodies in IgAN [44, 92].

## Soluble CD89 and circulating immune complexes

Another proposed explanation for the excessive formation of IgA1-containing immune complexes is an abnormal interaction between circulating IgA1 and the myeloid IgA receptor CD89 in IgAN [93]. CD89 is an Fc α receptor and exists in membrane-bound and soluble (sCD89) forms. Two isoforms of sCD89 have been described in vivo with the smaller isoform present in healthy subjects and IgAN, while the larger isoform is only present in the serum of patients with IgAN. It has been proposed that in IgAN, circulating polymeric IgA1-containing immune complexes induce cleavage and shedding of the extracellular domain of membrane-bound CD89, forming high molecular weight IgA1-CD89 complexes that are prone to mesangial deposition. Murine studies suggest that activation of mesangial cells by IgA1-containing immune complexes requires sCD89, a process that is also dependent on tissue transglutaminase 2 [94–96]. Recent data suggests that recurrent IgAN following transplantation is also associated with higher levels of IgA-sCD89 complexes [97]. There have, however, been conflicting studies reporting that sCD89-pIgA1 immune complexes are not specific or relevant to the development of IgAN [98–100].

## Mesangial deposition of immune complexes and triggering of glomerular injury

**Fig. 4:**
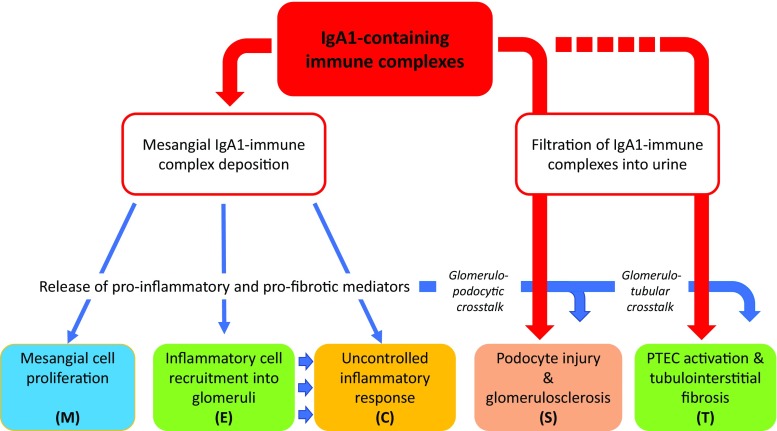
Pathological consequences of IgA immune complex deposition in IgAN. IgA1 immune complexes deposit in the mesangium and trigger mesangial cell activation, resulting in release of pro-inflammatory, chemotactic, and pro-fibrotic mediators. Released soluble mediators result in mesangial cell proliferation, extracellular matrix (ECM) synthesis, recruitment of inflammatory cells, and in severe cases, glomerular crescent formation. Filtered mesangial cell-derived mediators cause podocyte damage (glomerulopodocytic crosstalk) and with damage to the permselective glomerular basement membrane filtered IgA immune complexes compound podocyte injury. Filtered mesangial cell-derived mediators and IgA immune complexes are also capable of injuring proximal tubule epithelial cells (PTECs), promoting tubulointerstitial inflammation and scarring (glomerulotubular crosstalk). These pathogenic processes result in mesangial hypercellularity (M), endocapillary hypercellularity (E), segmental glomerulosclerosis (S), tubular atrophy/interstitial fibrosis (T), and crescent formation (C), pathological features that define the Oxford classification and have been shown to be independent predictors of outcome in IgAN

Increased levels of poorly *O*-galactosylated IgA1 and the production of *O*-glycan-specific antibodies result in the formation of IgA1-containing immune complexes that, in susceptible individuals, deposit in the mesangium and trigger glomerular injury. This deposition is thought to occur through a combination of mesangial trapping and an increased affinity of poorly galactosylated IgA1 for extracellular matrix components, such as fibronectin and type IV collagen [101, 102]. Once deposited, IgA1-containing immune complexes bind to and activate mesangial cells, leading to a wide range of molecular events, including the local production of cytokines, such as IL-6, tumor necrosis factor-α and transforming growth factor-β, promoting an inflammatory response, mesangial cell proliferation, extracellular matrix deposition, and in more severe cases glomerular crescent formation, driving glomerular and tubulointerstitial fibrosis (Fig. 4). These pathogenic processes correspond closely to the histopathological features identified in the Oxford classification, which are independent predictors of developing progressive renal disease in IgAN, namely mesangial hypercellularity (M), endocapillary hypercellularity (E), segmental glomerulosclerosis (S), and tubular atrophy/interstitial fibrosis (T), and as recently reported in an update to the original classification, glomerular crescent formation (C) [103, 104]. Mesangial cell IgA binding triggers the release of pro-inflammatory and chemotactic mediators, which act locally in the glomerulus, leading to mesangial cell proliferation (M) and recruitment of inflammatory cells into the glomerulus (E), occasionally resulting in crescent formation (C). These mediators also, in turn, alter podocyte gene expression and glomerular permeability, causing filtration of IgA immune complexes, podocyte damage (glomerulopodocytic crosstalk), and segmental glomerulosclerosis (S) [105–108]. In addition to effects within the glomerulus, glomerular-derived cytokines, along with filtered pIgA1, are capable of activating proximal tubule epithelial cells (glomerulotubular crosstalk), driving tubulointerstitial fibrosis (T) [109].

Recognition of mesangial IgA deposits by resident glomerular cells is incompletely understood. The best-characterized receptor for mesangial IgA is the transferrin receptor (CD71), which is expressed by mesangial cells. CD71 is a multi-ligand receptor that has been shown to bind polymeric IgA1 [110]. CD71 is overexpressed on the surface of proliferating human mesangial cells in IgAN, and co-localization of CD71 with IgA1 immune deposits has been demonstrated in kidney biopsies [111]. Furthermore, the binding of poorly *O*-galactosylated IgA1 to CD71 appears to further enhance the expression of CD71 on proliferating mesangial cells, creating an autoamplification loop for self-perpetuating glomerular injury [112]. It has been proposed that IgA binding to CD71 in IgAN also involves CD89, in that IgA1-sCD89 complexes are capable of binding to CD71 and activating mesangial cells [113]. However, glomerular deposition of CD89 has not been conclusively demonstrated in IgAN. Importantly, there is strong evidence that CD71 is not the only mesangial cell IgA receptor, however, none of the other well-characterized IgA receptors, including CD89, polymeric immunoglobulin receptor, and the hepatic asialoglycoprotein receptor, are expressed by human mesangial cells in health or in IgAN and the nature of this receptor(s) is not known [114].

Most studies examining renal injury in IgAN have focused on the effects of IgA on mesangial cell biology, however, with damage to the glomerular basement membrane, there is emerging evidence that IgA immune complexes can enter the urine and directly interact with other cells within the nephron [115]. Data supports a direct interaction between filtered IgA immune complexes and podocytes and proximal tubule epithelial cells [105, 109], resulting in podocyte injury and loss, and epithelial–mesenchymal transformation with consequent tubulointerstitial scarring, respectively. These effects appear to be specific to IgA immune complexes generated in IgAN and may be related, at least in part, to the poorly *O*-galactosylated hinge region of the IgA1 molecule. Understanding how filtered IgA immune complexes interact with podocytes and proximal tubule epithelial cells may help us understand why some patients with IgAN have mesangial deposition only, while others display marked podocyte injury and tubulointerstitial scarring, and why the degree of mesangial deposition does not correlate with the severity of ensuing renal inflammation and injury.

With the advent of a plethora of tyrosine kinase inhibitors, there is increasing interest in defining the intracellular biochemical pathways activated by IgA immune complexes in the kidney in IgAN. Spleen tyrosine kinase (Syk) signaling is of particular interest in IgAN as it is not only active in mesangial and proximal tubule epithelial cells but is also involved in immunoreceptor signaling in B cells and immunoglobulin production. Glomerular Syk phosphorylation is increased in rodent models of proliferative glomerulonephritis and correlates with serum creatinine and histological features of disease activity [116]. Inhibiting Syk signaling reduces pro-inflammatory cytokine production, tissue inflammation and damage in both in vivo and in vitro models of kidney injury [117, 118]. Kim et al. demonstrated a clear upregulation of glomerular phospho-Syk levels in IgAN [119]. In parallel, Syk inhibition was able to reduce the proliferative and pro-inflammatory effects of IgA immune complexes on mesangial cells in vitro, supporting the testing of Syk inhibition as a treatment for IgAN. SIGN (Syk Inhibitor in GlomeruloNephritis) is a currently open phase 2 randomized, double-blind, placebo-controlled trial that is examining the efficacy of fostamatinib, an oral selective Syk inhibitor, in IgAN (ClinicalTrials.gov Identifier: NCT02112838).

## The complement system in IgAN

There is strong evidence that glomerular injury in IgAN is associated with activation of the complement system [120]. Glomerular deposition of complement component 3 (C3) is commonly observed in kidney biopsies in the same distribution as IgA. The presence of C3, coupled with the near ubiquitous absence of C1q, is consistent with activation of the lectin and/or alternative pathways. Non-classical pathway complement activation is supported by the glomerular deposition of alternative pathway (properdin and factor H) [121, 122] and lectin pathway (mannan-binding lectin (MBL), MBL–associated serine proteases 1 and 2, and C4d) [123–125] components in IgAN. Furthermore, the presence of lectin pathway components C4d [126–128] and MBL [129] have been associated with increased disease activity and subsequent development of ESRD.

Genome-wide association studies in IgAN have identified a protective locus at chromosome 1q32 corresponding to deletion of *CFHR3,1* (*CFHR3,1Δ*). *CFHR3,1* encodes complement factor H-related proteins 3 and 1, regulatory proteins that compete with factor H for the binding of C3b. *CFHR3,1Δ* leads to uninhibited binding of C3b by factor H and more effective inhibition of the alternative pathway, hence providing a protective effect against alternative pathway activation in IgAN. Indeed, *CFHR3,1Δ* has been associated with higher levels of circulating complement factor H and a reduced level of complement activation split product C3a [130]. In addition, levels of circulating CFH correlate positively with circulating C3 levels and negatively with mesangial C3 deposition [130]. Histopathologically, *CFHR3,1Δ* is associated with reduced tubulointerstitial injury according to the Oxford classification criteria [131]. However, the precise molecular mechanism for this intriguing association in IgAN remains to be elucidated. A variable frequency of *CFHR3,1Δ* with opposing effects on different immune-complex associated diseases (*CFHR3,1Δ* is also protective against age-related macular degeneration but increases susceptibility to systemic lupus erythematosus and atypical hemolytic uremic syndrome) suggests that balancing selection exists in the expression of this allele, a hypothesis that remains to be tested.

Given the convincing evidence for complement activation in IgAN and the emerging availability of agents that selectively block complement activation, investigators are beginning to explore the utility of complement inhibition in IgAN. Eculizumab, a recombinant, fully humanized hybrid IgG2/IgG4 monoclonal antibody against complement C5, prevents the formation of membrane attack complex and has been shown to be effective in atypical hemolytic uremic syndrome and C3 glomerulopathy, glomerular diseases resulting from dysregulation of the complement system. There have been two case reports of eculizumab use in rapidly progressive IgAN. In both cases, eculizumab was associated with temporary benefit in stabilizing renal function or proteinuria, but in both cases, there was significant disease progression once eculizumab was discontinued [132, 133]. These case reports raise the possibility of complement inhibition as a potential future treatment strategy in IgAN. One agent under development is OMS721, a monoclonal antibody targeting mannan-binding lectin-associated serine protease-2 (MASP-2), the effector enzyme of the lectin complement pathway. Early data in IgAN suggest that OMS721, and inhibition of the lectin pathway, reduces proteinuria in IgAN. Further data are keenly awaited.

## Conclusions

There has been significant progress in our understanding of IgAN over the past decade, and key pathogenic changes have been identified. Central to our current understanding of the pathogenesis of IgAN is a greater awareness of the importance of IgA immune complexes and the role poorly *O*-galactosylated IgA1 and *O*-glycan-specific antibodies play in their formation. Mesangial immune complex deposition leads to mesangial cell proliferation and production of mesangial-derived mediators that drive podocyte and tubulointerstitial injury via mesangial-podocyte-tubular crosstalk. Critical questions, however, remain unanswered - the precise origins of poorly *O*-galactosylated IgA1 and *O*-glycan-specific antibodies are incompletely understood, as are the factors and mechanisms determining the nephritogenic potential of IgA1-containing immune complexes. Further advances in our understanding of the pathogenesis of IgAN will be crucial in the development of diagnostic and prognostic markers, and novel therapeutics to ameliorate disease progression.
